# Characteristics of tropical freshwater microalgae *Micractinium conductrix*, *Monoraphidium* sp. and *Choricystis parasitica*, and their potency as biodiesel feedstock

**DOI:** 10.1016/j.heliyon.2019.e02922

**Published:** 2019-12-05

**Authors:** Megga Ratnasari Pikoli, Arina Findo Sari, Nur Amaliah Solihat, Anita Herawati Permana

**Affiliations:** a Department of Biology, Faculty of Science and Technology, Universitas Islam Negeri (UIN) Syarif Hidayatullah Jakarta, Jl. Ir. H. Juanda No.95, Ciputat, Tangerang Selatan, Banten, Indonesia, 15412; b Center for Integrated Laboratory, Universitas Islam Negeri (UIN) Syarif Hidayatullah Jakarta, Jl. Ir. H. Juanda No.95, Ciputat, Tangerang Selatan, Banten, Indonesia, 15412; c Department of Quality Assurance of Food Industry, Politeknik AKA Bogor, Jl. Pangeran Sogiri No. 283, Bogor, Jawa Barat, Indonesia, 16154

**Keywords:** Biotechnology, Environmental science, Microbiology, Biodiesel, Bioenergy, Chlorophyta, *Choricystis*, Freshwater, Gintung, *Micractinium*, Microalgae, *Monoraphidium*, Tropical lake, Situ Gintung, Situ Pamulang

## Abstract

The depletion of fossil fuel reserves requires advance anticipation through the search for alternative energy from renewable natural resources. Microalgae have been known as potential organisms for biodiesel feedstock. However, in order to be developed on a large scale, microalgae must have superior traits so that further development becomes more comfortable and cheaper. Tropical lakes are a source of superior microalgae adapted to moderate conditions which can later save operational costs in large-scale production. Situ Gintung and Situ Pamulang are the two largest lakes in South Tangerang, Indonesia and are potential sources of microalgae. Four microalgae isolates from both lakes have been identified, and their potential has been examined. Within an observation period of 18 days, they showed similar growth patterns reaching more than 10^8^ cells mL^−1^ on day 14 and were able to resist increasing pH. The microalgae were identified through morphological observations and the sequencing of 23S rRNA genes with phylogenetic analysis. Each strain has a positive quality. Isolate G4-3, which was identified as *Micractinium conductrix,* and P5-4, which was identified as *Monoraphidium* sp., yielded biomass that exceeded 1.2 g L^−1^ with lipid content exceeding 60%. Likewise, G4-9, which was also identified as *Micractinium conductrix,* and P2-15, which was identified as *Choricystis parasitica*, have lipid content which accounted for 89.10% and 57.48%, respectively; although their biomass was lower. The percentage of fatty acid methyl esters of the four microalgae achieved >60–80%; thus, meeting the standard of biodiesel properties. Therefore, the microalgae isolates have great potential for being developed as biodiesel feedstock.

## 1 Introduction

The depletion of oil and coal reserves has motivated the development of biofuel from renewable resources. Among the biofuels, biodiesel has a very high energy balance compared to other alternative fuels. Biodiesel is produced primarily from lipids that are sourced from microorganisms, such as microalgae and cyanobacteria. It is widely known that tropical countries contain mega biodiversity of living things, including microalgae. As a tropical country, Indonesia has an abundant type of microalgae which have not been widely explored. Thus, biodiesel is a renewable alternative energy that looks promising for developing commercially in any country which still relies on petroleum today.

Microalgae have been known as lipid producers; this lipid can be used as feedstock for biodiesel production. The lipid content of microalgae reaches more than 50% of the biomass (Ghosh et al., 2016; Milano et al., 2016; Yee, 2016; Shuba and Kifle, 2018). In spite of the fact that the production of microalgae biomass is being done on an industrial scale, many factors still need to be considered. Certain strains have different requirements in terms of nutrient intake and supply, as well as environmental and culturing conditions, and lipid extraction techniques. As a result, numerous efforts have been undertaken by many studies. Optimization of culture conditions that mimic the site sampling conditions can be done to obtain high lipid-containing microalgae (Mutanda et al., 2011). Cultivation methods were used that utilize waste in order to obtain high biomass and lipids on a larger scale (Markou et al., 2014; Engin et al., 2018). Various methods of cultivating and harvesting microalgae biomass on a large scale have been established which can be generally classified into open or closed systems, and their selection is highly dependent on the type of microalgae strains (Milano et al., 2016). The search for reliable lipid extraction techniques has also been carried out to obtain high yields of fatty acid methyl esters. However, microalgal biodiesel still has many challenges. Among the various efforts that have been undertaken, biodiesel cultivation and conversion on a large scale have not yet met economic feasibility (Zhu et al., 2017), so that this study is still on-going.

Behind all these efforts, the search for superior strains is still relevant in order to achieve satisfactory production effectiveness. The key to the successful implementation of algae biodiesel at the commercial level includes the isolation and identification of high-yield strains that have fast growth rates (Ghosh et al., 2016). Bioprospecting of microalgae has led to the discovery of strains that mostly originate from certain groups of microalgae (Shuba and Kifle, 2018). The isolation of novel strains can be conducted by taking samplings from lakes that have not yet been extensively investigated (Lin et al., 2018). Developing tropical countries can search among their own natural resources, which provides benefits in terms of sustainable, inexpensive, and easy use while utilizing the given biodiversity. In addition, the moderate temperatures and abundant year-round sunlight are advantageous for the exploration of microalgae in the tropics.

Tangerang Selatan is an independent city covering an area of 147.2 km^2^, located in the southern part of the tropical island of Java, Indonesia. As a buffer zone for the capital city of Jakarta, Tangerang has nine lakes providing places for water infiltration. Situ Gintung (223,790 m^2^) and Situ Pamulang (127,474 m^2^) are the two largest lakes in Tangerang Selatan, and both have a history of being polluted. The local communities still keep their traditional culture of draining their wastewater into the lakes. According to data from 2013 and 2014 provided by the local environment agency, the lakes are medium-to heavily-contaminated, either in the inlet or the outlet parts (Sandy, 2015). Such organic pollution decreases the water quality; on the other hand, it provides a life for the community of microorganisms because of nutritional availability. However, the diversity of microalgae in both lakes has never been studied. Therefore, the current study has explored the diversity of microalgae in these lakes. The selected microalgae isolates, i.e. G4-3, G4-9, P2-15, and P5-4, were identified from their morphology and DNA sequences. Their growth, lipid content, and biodiesel properties were also examined. The main aim of this research is to prepare renewable resources for the future from local resources, in the form of lipid-producing microalgae that have the potential to be developed as biodiesel feedstock.

## 2 Materials and methods

### 2.1 Water sampling

The water samples were separately retrieved in the euphotic zones of Situ Gintung and Situ Pamulang. Using the purposive sampling method, the sampling area in each lake was determined by observing green surface water, and randomly spreading a 1 × 1 m^2^ plastic transect on five sites as replications. Each transect consisted of 5 random sampling points. Then a liter of water was collected from the 10–30 cm deep water column of each of the random sampling points. Light intensity, pH, salinity, and temperature were recorded on site using a water quality checker (Horiba, Japan), in addition to the site location.

### 2.2 Isolation and purification of microalgae

The water samples were serially diluted to 10^−1^-10^−3^ in sterile aquadest, and all the diluted solutions were inoculated to 40 mL BG-11 agar plates (1% agar) using the spread technique. BG-11 is a medium that is frequently used in the cultivation of green algae. After an incubation period of 2–3 weeks in a natural light-dark cycle, the growing colonies showing different morphology were separately enriched in a BG-11 liquid media and incubated for three weeks in a natural light-dark cycle. Then 1 mL of each of the enriched culture was serially diluted while being observed under a light microscope to monitor their uniformity. The diluted solutions were spread onto agar plates and incubated under the same conditions. The uniform growing colonies were purified using the streaking method and were finally preserved in a slant agar media as a pure culture (Waterbury, 2006).

### 2.3 DNA extraction and amplification

The morphology of the microalgae was observed under a light microscope and identified using identification keys. A molecular identification was also conducted. About 15 microalgae colonies were suspended with 250 µL lysis buffers containing 1% sodium dodecyl sulfate in 1.5 mL microtubes, then incubated in a water bath at 70 °C for 30 min while being vortexed 3–4 times for 10 s. A 0.1 mm diameter glass bead as large as the tip of the spatula was added to the cell suspension and was disrupted using a Genie disruptor (Scientific Industries, USA) at a speed of 2850 rpm for 1 min. A DNA binding buffer containing 200 μL of guanidine thiocyanate was added to the microtube and evenly mixed manually. The entire mixture was put into a spin column which had been embedded in the collection tubes and then centrifuged at a speed of 10000 xg for 1 min. The liquid stored under the column was discarded. The column containing DNA was washed twice with a 70% ethanol solution using the centrifugation procedure as above. The column was set in a new 1.5 mL microtube, 50 ul of 10 mM Tris HCl solution (pH 8.0) was added, and left at 70 °C for 5 min, and then centrifuged at a speed of 6000 xg for 1 min to elute DNA.

The DNA amplification was carried out by PCR using a pair of 23S plastid marker primers: p23SrV-fl 5′-GGACAGAAAGACCCTATGAA-3′ and p23SrV-rl 5′-TCAGCCTGTTATCCCTAGAG-3′, with product size of 410 nt (Sherwood and Presting, 2007). The PCR mixture used Gotaq Green Master Mix, following the provided protocol (Promega, USA). The PCR condition was 95 °C for 2 min for initial denaturation; 35 cycles of 94 °C for 20 s, 55 °C for 30 s and 72 °C for 30 s; and a final extension at 72 °C for 10 min, which was run using Proflex thermal cycler (Applied Biosystems, USA). The PCR product was sent to Macrogen (South Korea) to determine its nucleotide sequence.

### 2.4 Phylogenetic analysis

Phylogenetics of the microalgae was analyzed using the Maximum Likelihood method based on the Kimura 2-parameter model (Kimura, 1980) which was processed with the MEGA X program (Kumar et al., 2018). A tree that has the highest possible log (-1521.15) is displayed. Bootstrap values or percentage of trees showing tree topologies where related taxa gathered together are shown next to the branch. The initial tree (s) approach is estimated by the Maximum Composite Likelihood (MCL) approach, and then selecting the topology with the superior log-likelihood value. A discrete Gamma distribution was used to model the evolutionary rate of differences among sites (+G, parameter = 0.2949). A total of 27 nucleotide sequences were involved in the tree, with several substitutions per site. There are a total of 369 positions in the last data set after all positions containing gaps and missing data were removed.

### 2.5 Examination of microalgae growth

The microalgae were grown in a BG-11 media from a stock culture. The volume of the culture was increased gradually. The first culture was started by inoculating five colonies from the stock culture into a 5 mL of media. The culture was shaken manually twice a day, once in the morning and once in the evening, for two weeks. All the cultures had natural lighting for 12 h a day at a room temperature of approximately 30 °C. In the second stage, the culture from the first stage was inoculated into 100 mL of fresh media and cultivated by aeration for two weeks, using a set of aerators. Finally, in the third stage, the culture from the second stage was inoculated into a liter of media and incubated under the same conditions as the previous stages. Each culture was sampled for cell enumeration and pH measurement at 1–2 day intervals. The cell enumeration was carried out using a Neubauer cell counter under a light microscope.

### 2.6 Direct transesterification

The direct transesterification method followed other methods (Johnson and Wen, 2009) with slight modifications. The biomass was concentrated by filtration, dried using oven-heating (60 °C) overnight, and the biomass yield was analyzed gravimetrically. About 0.5 g of the microalgal dry biomass was mixed with 1 mL of solvent. The solvent composition was methanol-sulfuric acid (6:1) and chloroform, with a ratio of 1:1. The biomass was crushed with the solvent and then transferred to a test tube while adding 2 mL of the solvent mixture. The tube was covered with a marble and heated at 70 °C for 2 h in a water bath. After the reaction was complete, the tube was left to cool until it reached room temperature. Then 2.5 mL aquadest was added to the mixture, homogenized with a vortex for 10 s, 2 mL of chloroform and 0.5 mL of aquadest were added, and homogenized again for 10 s. The mixture was then centrifuged at 6000 rpm for 10 min. The chloroform phase in the bottom layer was carefully decanted and transferred to a weighed glass tube. The phase containing the lipid was dried in an oven at 60 °C for about 4 h which was interspersed by weighing at intervals until the weight was constant. The microalgae with the highest yield were selected for determination of the fatty acid methyl ester (FAME) using GC-MS.

### 2.7 Gas chromatography - mass spectrometry (GC-MS)

Analysis of the direct transesterification products was carried out by using GCMS-QP2010 (Shimadzu). The initial temperature was set at 60 °C and fixed for 1 min. The final temperature was set at 250 °C, with a temperature increase of 7 °C per minute. The final temperature was fixed for 10 min, followed by 3 min of equilibration time. Helium gas was used as a carrier with a flow rate of 1.00 mL per minute. The four samples were analyzed under identical conditions. Each peak that emerged from gas chromatography was analyzed by electron ionization-mass spectrometry (EI-MS). Data from the GC separation and MS fragmentation were processed using GCMSsolution Ver software. 4.

### 2.8 Determination of biodiesel properties

The biodiesel properties include the average degree of unsaturation, kinematic viscosity, specific gravity, cetane number, iodine value, and higher heating value. The average degree of unsaturation (ADU) values from microalgae lipids were determined using the equation below (Hoekman et al., 2012):

$$\text{ADU}={\sum \text{M}\times Y_{i}}^{}$$

in which M is the number of carbon-carbon double bonds in each fatty acid constituent, and ${\text{Y}}_{\text{i}}$ is the mass fraction of each fatty acid constituent. Data sources for M and ${\text{Y}}_{\text{i}}$ were obtained from GC results. There is a correlation between ADU value and other parameters, which are shown in the equations:

Kinematic viscosity, Vis=−(0.6316×ADU)+5.2065

Specific gravity, SG=(0.0055×ADU)+0.8726

Cetane number, CN=−(6.6684×ADU)+62.876

Iodine value, IV=(74.373×ADU)+12.71

Higher heating value, HHV=(1.7601×ADU)+38.534

The obtained properties of FAME were compared to those from other studies, with the reference standard of ASTM D6751 (ASTM, 2008) and EN 14214 (European Committee for Standardization, 2008).

## 3 Results

### 3.1 Physical and chemical conditions of the lakes

Situ Gintung and Situ Pamulang have an area of 21 ha and 8 ha with a shallow depth of 10 m and 8 m, respectively. The water sampling was conducted within the area of 06°18′36.4–28.5″S, 106°45′54.8–48.2″E in Situ Gintung, and 06°20′01.7–59.0″S, 106°44′39.0–36.3″E in Situ Pamulang. The total dissolved solids, pH, dissolved oxygen, and salinity of the water of the two lakes showed values that met the standards of freshwater use for fisheries and gardening requirements as established by the Indonesian government regulations. The most contrasting difference between the two lakes was that the turbidity of Situ Gintung water was almost twice as high as that of Situ Pamulang. High light intensities are in accordance with the level of solar irradiation in the tropics; namely, for about 5 h a day at 09–14 Western Indonesia Time, which is the optimum time range for solar radiation in this region. The water conditions (Table 1) at the time of samplings provided information about the environmental conditions of the microalgae in their natural habitat, which was partially used in their cultivation.

**Table 1:** Physical and chemical conditions of water in both lakes.

Parameters	Situ Gintung	Situ Pamulang
Water temperature (°C)	34.05 ± 1.45	32.73 ± 0.50
Air temperature (°C)	36.50 ± 4.14	33.05 ± 0.54
Light intensity (Lux)	74225 ± 13705	14018 ± 1735
Total dissolved solids (TDS, ppm)	96.50 ± 14.64	108.00 ± 1.15
pH	7.59 ± 0.31	7.21 ± 0.10
Dissolved oxygen (mg/L)	6.68 ± 1.43	6.82 ± 0.61
Salinity (%)	0.01	0.01
Turbidity (NTU)	112.80 ± 16.53	61.63 ± 12.24

### 3.2 Results on the identification of microalgae

**Figure 1:**
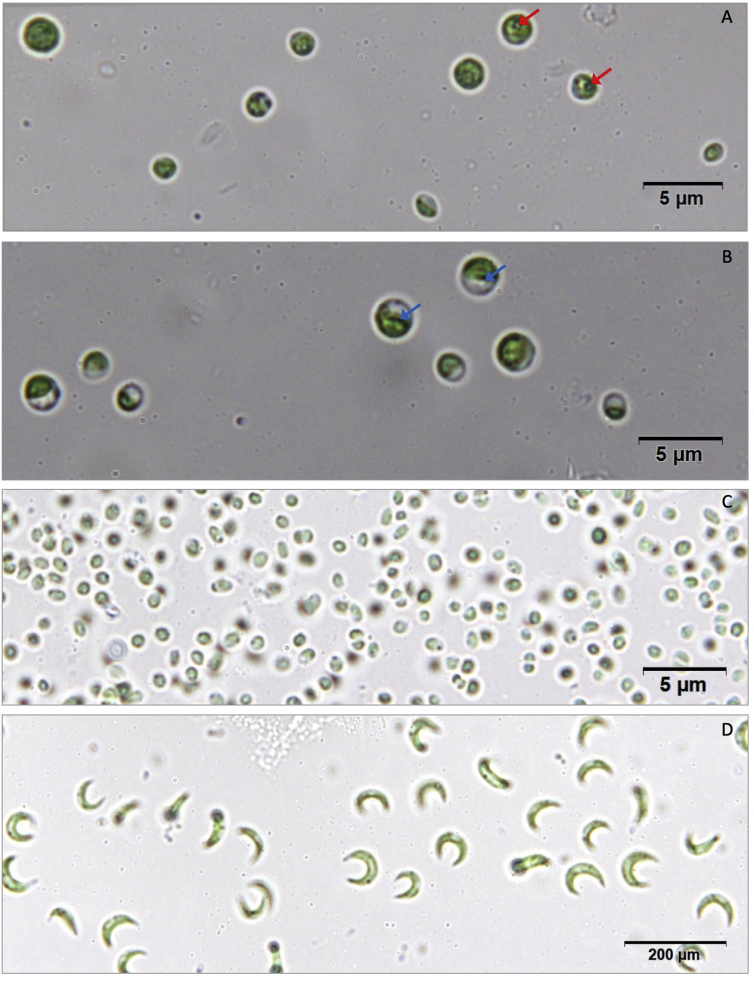
Photomicrograph of microalgal cells. A. Isolate G4-3 (red arrow = pyrenoid); B. Isolate G4-9 (blue arrow = parietal); C. Isolate P2-15; D. Isolate P5-4.

**Figure 2:**
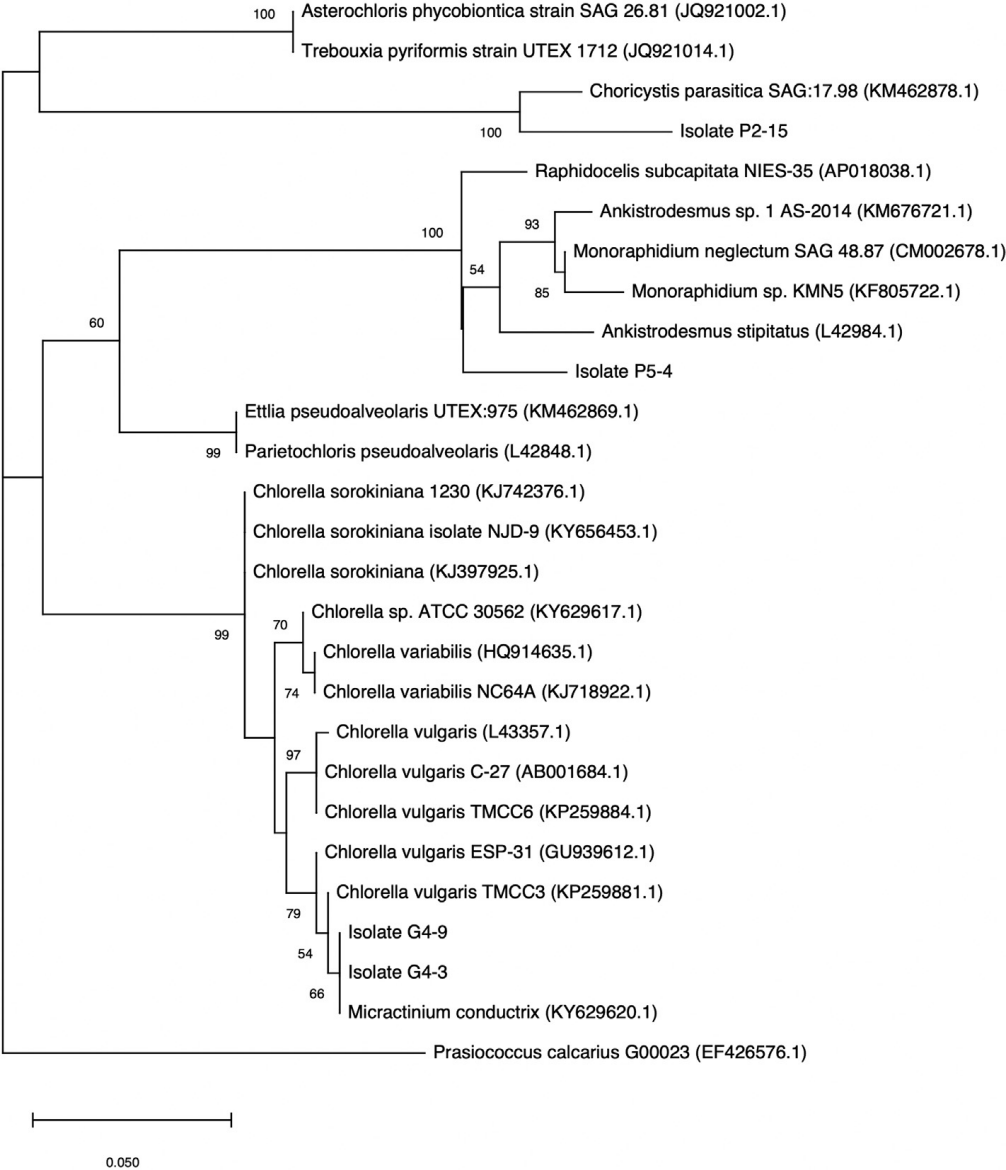
Phylogenetic tree of the four isolates together with reference sequences that retrieved from genbank NCBI. *Prasiococcus calcarius* is used as the outgroup. Bootstrap replication 1000 times.

The identification of the four microalgae began with observations of their cell morphology. The results showed that they have different morphologies (Figure 1). G4-3 and G4-9 have the same form: solitary living cells, spherical, smooth walls, and 2–4 μm cell diameters. Such cell morphology shows that they are both *Chlorella-like* cells. The two isolates seemed similar, even though they were not. The difference is indicated by the presence of pyrenoid in the G4-3 cells and parietal chloroplast in the G4-9 cells. The identification results from the cell morphology were then verified by molecular identification through sequencing of the plastid 23S rRNA genes and alignment with sequences from the genebank (NCBI), and the results showed that they are closest to *Micractinium conductrix* (Figure 2). P2-15 is characterized by tiny cells with a size of 0.5–1 μm in width, and are spherical to ellipsoidal (Figure 1C). The analysis results of the plastid 23S rRNA genes showed a 100% similarity and a bootstrap value of 100 to *Choricystis parasitica* (Figure 2). The morphology of the crescent-shaped P5-4 cells (Figure 1D) has been directed to the Selenastraceae group, and the 23S rRNA genes phylogenetic tree has verified this (Figure 2). The gene sequences amplified from the four microalgae were deposited in NCBI with accession numbers MH795193-MH795196. Henceforth, the results of the identification of the four microalgae will be used in the results and subsequent discussion in this paper.

### 3.3 Growth of microalgae

**Figure 3:**
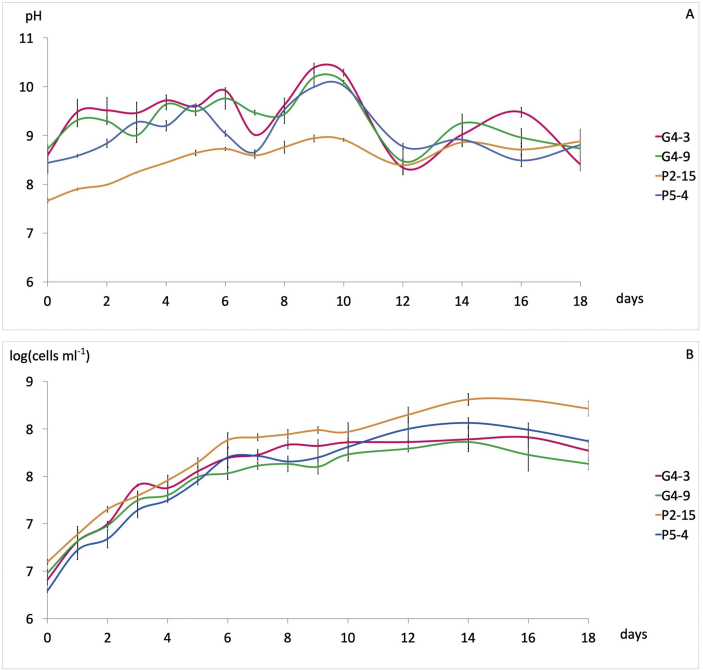
Results on microalgal culture examination. A. pH; B. cell concentrations.

During the 18 days of microalgae growth in the BG-11 media, there were slight fluctuations in the pH (Figure 3A). The pH was in the range of 8–10, with an average of 9.07, with the initial pH of the media around 7.4 before being inoculated; no adjustment was made to determine the differences between the cultures. Shortly after being inoculated, the initial pH of the four microalgae looked similar; i.e., 8.44–8.73, except for the lower *C. parasitica* P2-15 (average 7.66). Likewise, the pattern of pH fluctuations from the four microalgae looked similar, except for *C. parasitica* P2-15. This is thought to be influenced by the pH of the inoculum culture. The lower pH of *C. parasitica* P2-15, as compared to the other isolates, can be the characteristic of this isolate. *M. conductrix* G4-3 and G4-9 have the most similar pH fluctuations, which is thought to be due to the close relationship between the two strains.

Since the initial incubation growth period, the growth of all the microalgae increased immediately; there was no apparent adaptation phase (Figure 3B). The peak exponential phase occurred on the 14^th^ day of incubation and then entered the stationary phase. Growth then tended to slow down until the end of the observation period. *C. parasitica* P2-15 appeared to be superior in its growth, while *M. conductrix* G4-3 and G4-9 growth were the lowest. Another matter of concern is the existence of three growth sequences; namely, days 0–6, 6–14, and 14–18. The fastest growth rate or exponential phase seems to occur from the beginning of the incubation until day 6. The absence of a lag phase shows that the previous three stages of activation succeeded in reducing the adaptation phase in all the cultures; namely, the cells grow immediately once they are in the fresh medium. Days 6–14 is a stationary phase which is characterized by a slow growth rate. This long stationary phase is thought to be associated with sharper pH fluctuations, which reached peaks on the 9^th^ day. Furthermore, growth tended to decline from days 14–18, which indicates that the environmental conditions in the medium no longer supported growth.

### 3.4 Yield of microalgae and biodiesel properties

**Figure 4:**
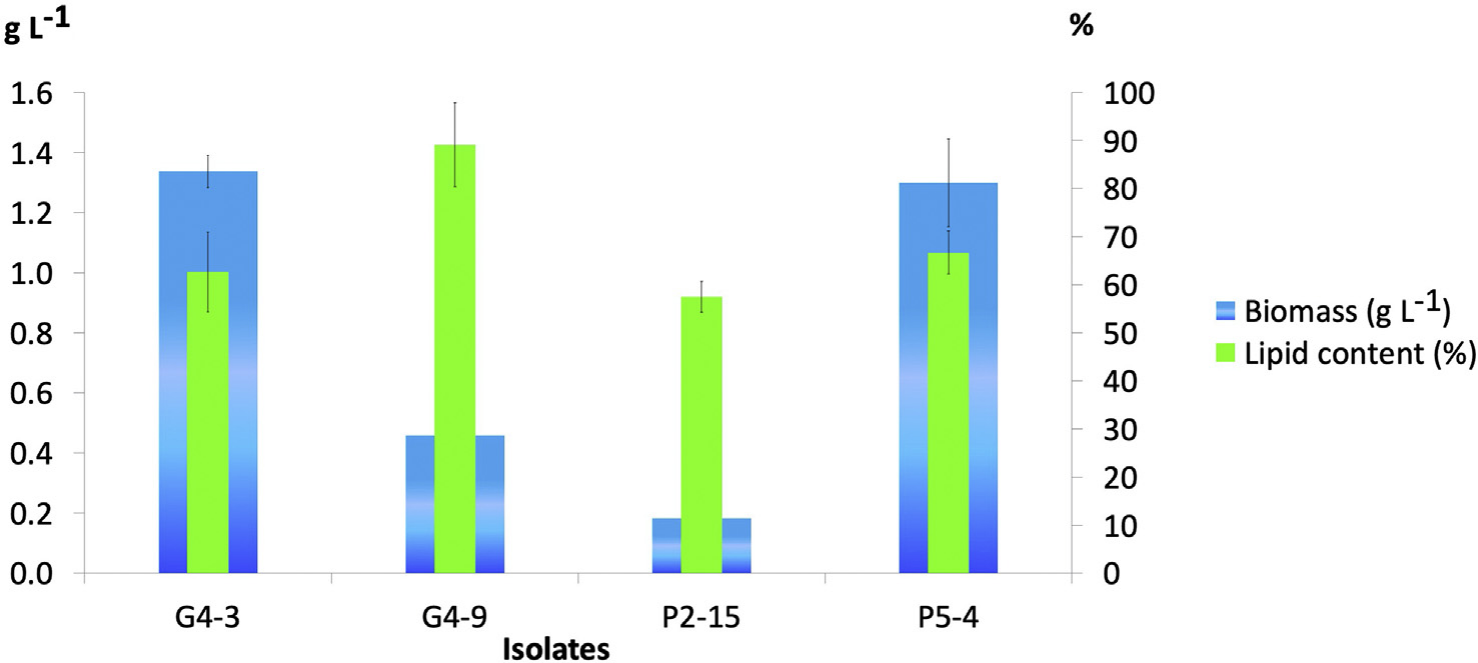
Yield of biomass and lipid content.

Yield of microalgae as a feedstock for biodiesel can be considered from the production of the biomass and the lipid content. The four microalgae showed potential, both as far as their biomass and their lipid content (Figure 4). *M. conductrix* G4-3 has the highest dry biomass of 1.34 ± 0.05 g L^−1^. The microalgae with the next highest biomass is *Monoraphidium* sp. P5-4 with a yield of 1.30 ± 0.15 g L^−1^ and a lipid content which reached 66.72 ± 4.52% or 4.52 ± 0.007 g L^−1^. At the same time, the yields of *C. parasitica* P2-15 and *M. conductrix* G4-9 were lower than the other two microalgae.

The total FAME of the direct transesterification of fatty acids resulting from the four microalgae had a high percentage (above 60%) of total lipids (Table 2). G4-3, P2-15, and P5-4 have variations of fatty acids with C14 – C24 chain lengths, while G4-9 has variations of C14 – C18 fatty acids (Table 2). In general, fatty acids from the four microalgae of Situ Gintung and Pamulang Situ were dominated by C18 and C16 chains. The dominant FAME component produced by the *M. conductrix* G4-9 and *Monoraphidium* sp. P5-4 was methyl *α*-linolenate (C18:3n-3) in the amount of 23.95% and 32.00%, respectively. The dominant FAME of *M. conductrix* G4-3 was methyl linoleate (C18:2n-6) which accounted for 20.24%. Differing from the other three microalgae which produced the dominant FAME in the form of C18 chains, *C. minor* P2-15 produced methyl palmitate (C16) which accounted for 29.11% (Table 2).

**Table 2:** Composition of FAME resulted from microalgae.

Fatty acid	FAME	% of total lipid			
		G4-3	G4-9	P2-15	P5-4
C14	Methyl myristate	0.45	0.33	4.29	0.43
C15	Methyl pentadecanoate	0.47	-	-	0.65
C16	Methyl palmitate	4.22	15.34	29.11	9.18
C16:1n-7	Methyl palmitoleate	3.98	2.12	1.55	3.38
C16:2n-6	Methyl 7,10-hexadecadienoate	3.89	7.23	0.78	2.39
C16:3n-3	Methyl 7,10,13-hexadecatrienoate	3.17	11.07	-	0.73
C16:3n-6	Methyl 4,7,10-hexadecatrienoate	0.78	-	-	3.45
C17	Methyl margarate	-	-	-	0.37
C18	Methyl stearate	-	-	0.98	1.21
C18:1n-4	Methyl 14-octadecenoate	-	1.77	-	0.61
C18:1n-7	Methyl 11-octadecenoate	-	-	1.37	-
C18:2n-6	Methyl linoleate	20.24	21.65	4.95	15.55
C18:3n-3	Methyl *α*-linolenate	14.82	23.95	26.83	32.00
C18:3n-6	Methyl *γ*-linolenate	1.04	-	1.46	0.87
C20	Methyl arachidate	-	-	-	1.04
C20:1n-9	Methyl 11-eicosenoate	-	-	0.94	-
C20:4n-3	Methyl 5,11,14,17-eicosatetraenoate	10.80	-	-	10.15
C20:5n-3	Methyl eicosapentaenoate	1.58	-	4.18	3.25
C22	Methyl behenate	-	-	-	0.33
C24	Methyl lignocerate	0.95	-	3.88	0.45
C24:1n-9	Methyl 15-tetracosenoate	-	-	0.35	-
Total rendemen (%)		**66.39**	**83.46**	**80.67**	**86.04**

The four microalgae have values that are close to the standard for biodiesel (Table 3), although there are some weaknesses. *M. conductrix* G4-3 and G4-9 have a weakness in iodine value (IV), which exceeded the standard. Besides IV, *Monoraphidium* sp. P5-4 also has an average degree of unsaturation (ADU) and higher heating value (HHV) that exceeds the standards. At the same time, *C. parasitica* P2-15 met all the criteria of the standards.

**Table 3:** Properties of FAME produced by the microalgae.

Isolate	ADU	Vis, at 40 °C (mm^2^ s^−1^)	SG (kg L^−1^)	CN	IV (g I_2_/100 g)	HHV (MJ kg^−1^)
*Micractinium conductrix* G4-3	1.63	4.18	0.882	52.0	133.94	41.4
*Micractinium conductrix* G4-9	1.67	4.15	0.882	51.7	136.91	41.5
*Choricystis parasitica* P2-15	1.21	4.44	0.879	54.8	102.70	40.7
*Monoraphidium* sp. P5-4	2.08	3.89	0.884	49.0	167.41	42.2
Biodiesel standard	0.6–1.6	1.9–6.0∗	0.85–0.90∗	≥47∗	≤120∗∗	38–41

∗ US (ASTM D6751-08) standard.

∗∗ Europe (EN 14214) standard.

## 4 Discussion

Situ Gintung and Situ Pamulang had air temperatures and surface water above 30 °C, which is characteristics of tropical lakes that have an average temperature above 4 °C. Although the water temperature exceeded the optimum temperature range for the growth of various algal species; namely 20–30 °C (Singh and Singh, 2015), the temperature did not reach 40 °C. Temperatures above 40 °C will inhibit the charge-separation activity of photosystem II and thus, impede photosynthesis (Ras et al., 2013).

The light intensity at Situ Pamulang at the time of the samplings was 322 μmol m^−2^ s^−1^ which was still below the maximum limit of 420 μmol μmol m^−2^ s^−1^ for algal growth which has been reviewed by others (Singh and Singh, 2015). However, the light intensity at Situ Gintung was very high; namely, 1707 μmol m^−2^ s^−1^. About 65 different colonies were obtained from Situ Gintung. This number is higher than those obtained from Situ Pamulang (48 different colonies). This shows that the high light intensity is not a limiting factor in the diversity of microalgae types in these lakes. Various microalgae in this environment have adapted to the high sunlight intensity.

In general, other parameters of water in both lakes, such as total dissolved solids and turbidity, indicated environmental conditions that support microalgae growth. The total dissolved solids can describe dissolved inorganic minerals that originate from the decomposition of organic materials which support microalgae growth. Turbidity is thought to reflect the high density of the plankton community, including microalgae, because some water samples were light green. The high turbidity at Situ Gintung can, in turn, provides shading for the microalgae community on the water surfaces of the lakes from the high light intensity. Biomass of microalgae itself, as well as other organic materials in the water, hinder the overexposure of sunlight. Conditions of the sampling environments can be used as underlying conditions for *in vitro* cultivation of the microalgae (Mutanda et al., 2011).

Morphologically, both G4-3 and G4-9 resemble *Chlorella*, of which G4-3 has pyrenoid, whereas G4-9 does not. The presence of pyrenoid in spherical cells without any splits shows characteristics of *Chlorella vulgaris*, while cells that do not show a pyrenoid of this size characterize *Chlorella minuttissima* (John and Tsarenko, 2002). The phylogenetic tree reconstructed from the plastid 23S rRNA genes indicates that the G4-3 and G4-9 are in one clade along with various types of *Chlorella* (Figure 2). However, the closest relationship with 66% bootstrap value and 100% blast similarity was with *Micractinium conductrix*. Compared with *Chlorella*, information about the plastid 23S rRNA genes of *Micractinium*, especially *M. conductrix,* is very limited.

*Micractinium* is a *Chlorella-like* genus, which has spherical-to-ovoid cells and possesses a parietal and cup-shaped chloroplast with a pyrenoid (Hong et al., 2015). Traditionally, the morphology of *Micractinium* is characterized by the presence of spines or bristles that protrude from the cell wall with cell formations like that of *Chlorella* (John and Tsarenko, 2002)*.* However, G4-3 and G4-9 did not show the presence of any spines or bristles (Figure 1). The fact that not all *Micractinium* have bristles is known from other studies (Krienitz and Bock, 2012; Luo et al., 2010; Pröschold et al., 2010; Hong et al., 2015). This property is similar to *Micractinium* KNUA029 and 034 strains isolated from Antarctica; they do not have bristles but have phylogenetic proximity with strains that form bristles and accumulate in *Chlorella* clade, based on SSU and ITS sequences of nuclear-encoded rRNA gene (Hong et al., 2015). The ITS sequences of the two microalgae were also examined in this research, but the resulting phylogenetic tree did not separate *Chlorella* and *Micractinium* (data not shown). Another study also failed to use the ITS region for the majority of Chlorophyta isolates examined (Vieira et al., 2016).

Bristles are thought to be a form of adaptation to grazing pressures by zooplankton. This is demonstrated in a study in which an environment without pressure did not cause *Micractinium* to form bristles (Luo et al., 2010). Other researchers proposed the separation of *Micractinium* which have bristles from those who do not (Proeschold et al., 2011). Micractiniaceae were traditionally considered as one of the Chlorococcales with bristles. Based on the SSU and ITS sequences, *Micractinium,* are indeed, closely related to *Chlorella*, but only a few form bristles, such as *Micractinium pusillum* and *M. parvulum*. *Micractinium* usually form colonies and have bristles, but their members disintegrate to form cells without bristles (Krienitz and Bock, 2012).

Morphologically, it is difficult to determine the identity of tiny cells of P2-15 because observations using the light microscope have their limitations. The difficulty in identifying P2-15 based on its cell morphology is convincingly surmounted by the phylogenetic tree that showed high similarity to *Choricystis parasitica* (Figure 2). In contrast to the identification of the three microalgae, molecular identification of P5-4 requires verification of morphological characteristics.

In the phylogenetic tree (Figure 2) P5-4 was in a clade containing the Selenastraceae group with proximity to *Monoraphidium* and *Ankistrodesmus*. Although it is not clear to which genus it is most closely related/belongs to, *Ankistrodesmus* or *Monoraphidium*, the cell structure, and size are large enough to suggest that this isolate is *Monoraphidium* sp. The cells are markedly curved or crescent-shaped, and most have pointed ends. Such cell morphology with a length of about 100 μm resembles that of *M. mirabile.*

During the cultivation of microalgae, there was a slight fluctuation in pH (Figure 3A). The pH fluctuated due to the photosynthetic activity which converts bicarbonate to carbon dioxide to produce OH^−^ which increases the pH of the medium, which the cells then neutralize by capturing H^+^ from the surrounding environment (Markou et al., 2014). Other studies support that the genera are able to tolerate pH 9–10 well, for example *Choricystis minor* (Sobczuk and Chisti, 2010) and *Micractinium* sp. (Skrupski et al., 2013).

The 18-day growth pattern of the four microalgae appeared to be similar (Figure 3B). It seems that this growth sequence is related to pH in which the closer the pH to 7.2–7.5 (the pH of the sample water), the better the growth. The growth rate is influenced by the pH of the medium where the cell growth depends on the pH of the environment from which the microalgae is isolated. For instance, microalgae isolated from alkaline environments are able to reach the highest number of cells at high pH (Gardner et al., 2011).

The yield of the microalgae isolates resembles the maximum gain obtained from other studies. *M. conductrix* G4-3 that produced dry biomass 1.34 ± 0.05 g L^−1^ resembles *Micractinium pusillum* that produced the highest 1.28 ± 0.04 g L^−1^ with lipid content of 0.47 ± 0.05 g L^−1^ (Abou-Shanab et al., 2012), and *Micractinium* sp. ME05 with a maximum yield of 1.38 ± 0.095 g L^−1^ for seven days with lipid content of 7.21 ± 0.44% (Engin et al., 2018). The yield from *M. conductrix* G4-3 was higher than that of *Micractinium resseri* JN 169781 which was 0.75 ± 0.014 g L^−1^ on day 7 with lipid content of 26.5 ± 1.1% (Wang et al., 2018). Moreover, the lipid content from *M. conductrix* G4-3 and G4-9, which was 62.63 ± 8.32% and 89.10 ± 8.72% respectively, was higher than other strains. *C. parasitica* P2-15 produced lower biomass, i.e. 0.18 ± 0.04 g L^−1^. This yield is much lower than *C. minor* from another study that reached 2.02 g L^−1^, with lipid content of 21.3 ± 1.7% (Sobczuk and Chisti, 2010). Nevertheless, its lipid content is still very high that 0.18 ± 0.04 g L^−1^ of biomass contained 57.48 ± 3.19% lipid. Productivity or acquisition of biomass and lipid content can be affected by changes in the pH. Changes in pH are responded to differently by different species; some respond with increased productivity of biomass to increasing pH, and some respond otherwise; but the stress of increasing pH does cause increased production of triacylglycerol (Skrupski et al., 2013).

The high potential of the biomass and lipid content of *Monoraphidium* have been widely studied, and it was concluded that Selenastraceae is an emerging candidate for biodiesel production (Yee, 2016). *Monoraphidium* sp. P5-4 on day 18 can be said to be higher than that obtained from *Monoraphidium* HDMA-11 which obtained 0.567 g L^−1^ and lipid content 28.5% (Lin et al., 2018). The yield of these microalgae is still lower than that of *Monoraphidium minutum* which has productivity of 0.3038 g L^−1^ day^−1^ (or accounting for 5.468 g L^−1^ after 18 days) (Patidar et al., 2014), *Monoraphidium* sp. Deck19 with a yield of 1.52 g L^−1^ (Holbrook et al., 2014), *Monoraphidium* sp. ABC-02 with a yield of 3.86 ± 0.305 g L^−1^ (Shrivastav et al., 2015), and *Monoraphidium* sp. QLY-1 with a yield of 5.54 g L^−1^ (Zhao et al., 2016). Nevertheless, the lipid content of *Monoraphidium* sp. P5-4, which exceeds 60%, was higher than the other microalgae, indicating that it also has the potential to be a superior isolate for biodiesel feedstock.

The FAME recovery of *Monoraphidium* sp. P5-4 is similar to that produced by *Monoraphidium* sp. ABC-02 which accounted for 79.21% using sodium nitrate as a source of nitrogen (Shrivastav et al., 2015), and *Monoraphidium* QLY-1 which accounted for 70.49% (Zhao et al., 2016). *C. parasitica* P2-15, with 80.67% FAME, is similar to *C. minor* which produced 84.1 ± 6.3% FAME (da Cruz Lima et al., 2018). At the same time, the percentage of FAME (66.9%) from *M. conductrix* G4-3 needs to be increased so that it can reach more than the 94% FAME which is produced by *Micractinium* sp. ME05 (Engin et al., 2018).

Types of fatty acids produced by the four microalgae isolates are following the nature of common microalgae, which generally provides fatty acids with chain lengths of C16 and C18 (Breuer et al., 2013). Fatty acids commonly found in microalgae (C16–C18) are suitable for biodiesel production; namely palmitic (16:0), stearic (18:0), oleic (18:1), linoleic (18:2) and linolenic (18:3) *acids* (Knothe, 2009; Abou-Shanab et al., 2012; Engin et al., 2018).

The high IV of *M. conductrix* G4-3 and G4-9 is often associated with oil properties which are more susceptible to oxidation. However, low IV due to low molecular weight does not affect the oxidative stability of double bonds in fatty acid chains, so IV does not have to be considered in the selection of optimal biodiesel components (Knothe, 2009). The high ADU of *Monoraphidium* sp. P5-4 is due to its composition which is dominated by unsaturated fatty acids. Similarly, HHV, which is slightly higher than the standard, is caused by having more double bonds so that it requires more energy. Although HHV is slightly above the standard, the value of 42.2 MJ kg^−1^ is still within the range of 29–45.9 MJ kg^−1^ which is a typical value for microalgal oil (Brennan and Owende, 2010). Although the biomass yield of *C. parasitica* P2-15 was not satisfactory when compared to the other microalgae, the percentage of FAME which reached 80.67% with the dominant composition of C16 (29.11%) and C18:3n-3 (26.83%) is a better source of biodiesel feedstock. In the future, it will be necessary to find a method and cultivation media that can optimize the amount of biomass yield.

In conclusion, the four microalgae isolated from Situ Gintung and Situ Pamulang, the two tropical lakes in South Tangerang, Indonesia, have advantages in terms of growth, biomass yield, lipid content, percentage of fatty acid methyl esters, and biodiesel properties. The advantages of the *Micractinium conductrix* G4-3, *Micractinium conductrix* G4-9 and *Monoraphidium* sp. P5-4 mainly lie in the biomass yield and lipid content, while the advantages of *Choricystis parasitica* P2-15 are its methyl palmitate component and biodiesel properties. Therefore, the four microalgae show potential as biodiesel feedstock which needs to be optimized in the future.

## Declarations

### Author contribution statement

Megga Ratnasari Pikoli: Conceived and designed the experiments; Performed the experiments; Analyzed and interpreted the data; Contributed reagents, materials, analysis tools or data; Wrote the paper.

Arina Findo Sari: Performed the experiments; Contributed reagents, materials, analysis tools or data.

Nur Amaliah Solihat: Performed the experiments.

Anita Herawati Permana: Analyzed and interpreted the data.

### Funding statement

This work was supported by the Kuasa Pengguna Anggaran No. Un.01/KPA/574/2017, UIN Syarif Hidayatullah Jakarta, Kementrian Agama Republik Indonesia.

### Competing interest statement

The authors declare no conflict of interest.

### Additional information

Data associated with this study has been deposited at GenBankNCBI under the accession numbers MH795193-MH795196.
